# Novel imaging modalities for the identification of vulnerable plaques

**DOI:** 10.3389/fcvm.2024.1450252

**Published:** 2024-09-12

**Authors:** Ziyan Wang, Pingyang Zhang

**Affiliations:** Department of Cardiovascular Ultrasound, Nanjing First Hospital, Nanjing Medical University, Nanjing, China

**Keywords:** atherosclerosis, vulnerable plaque, computed tomography, magnetic resonance, ultrasound, positron emission tomography, optical coherence tomography

## Abstract

Atherosclerosis is a slow, progressive disease that is closely associated with major adverse cardiovascular events. Early diagnosis and risk assessment of atherosclerosis can effectively improve the prognosis and reduce the occurrence of adverse cardiovascular events in the later stage. A variety of invasive and non-invasive imaging modalities are important tools for diagnosing lesions, monitoring the efficacy of treatments, and predicting associated risk events. This review mainly introduces the four commonly used non-invasive imaging modalities in clinical practice and intravascular imaging such as optical coherence tomography, intravascular ultrasound imaging, and near-infrared spectroscopy, compares the advantages and disadvantages in the diagnosis of vulnerable plaques, and briefly summarizes the new progressions of each.

## 1 Introduction

Atherosclerosis, a chronic, multifactorial, complex disease, is the primary cause of a life-threatening cardiovascular disease, which causes much morbidity, hospitalization, and mortality worldwide (1). After decades of silent progression, atherosclerotic plaque rupture with thrombus formation is the principal pathophysiological mechanism responsible for the development of acute coronary syndromes (1, 2). Traditionally, the clinical assessment of atherosclerosis focuses on the degree of luminal narrowing. As the concept of the “vulnerable plaque” or “unstable plaque” is gradually recognized, it is more comprehensive to identify patient “cardiovascular vulnerability” that inclines to direct assessment of the plaque or indirect statistical risk scores through the morphological and hemodynamic factors in the clinical practice.

The vulnerable plaque is commonly understood as the plaque with specific structural characteristics such as thin fibrous caps, large lipid cores, positive vascular remodeling, intraplaque hemorrhage, macrophage infiltration, and neovascularization (3, 4). Conventional catheter-based coronary angiography is the gold standard that provides direct visualization of the degree of luminal narrowing. However, it fails to assess the hemodynamic consequences of stenoses and the accurate structural features under the endothelium (4). In recent years, several imaging modalities with varying sensitivities and specificities have emerged as the times require. This review aims to summarize the novel imaging modalities for the assessment of vulnerable plaque and prediction, focus on advantages and limitations respectively, and discuss the prospect in the early diagnosis of atherosclerotic vulnerable plaques.

## 2 Non-invasive imaging

Non-invasive imaging, like computed tomography (CT), magnetic resonance (MR), non-invasive ultrasound techniques, and positron emission tomography (PET), contributes to estimative risk stratification and prediction in low-intermediate-risk populations.

### 2.1 Computed tomography (CT)

CT is a common diagnostic imaging method used for the examination of various diseases with the characteristics of fast scanning time and clear images. Apart from functional assessment by CT like myocardial CT perfusion or non-invasive CT-derived fractional flow reserve that concentrates on hemodynamically significant stenoses (5), CT imaging can easily detect calcification, which leads to a direct quantitative assessment of coronary artery calcium score (CACS) (4, 6). The high-risk plaque features increase as the Agatston CACS increases (7). Nevertheless, the prognosis is not perfect for patients with zero or minimal calcium score.

**Figure 1:**
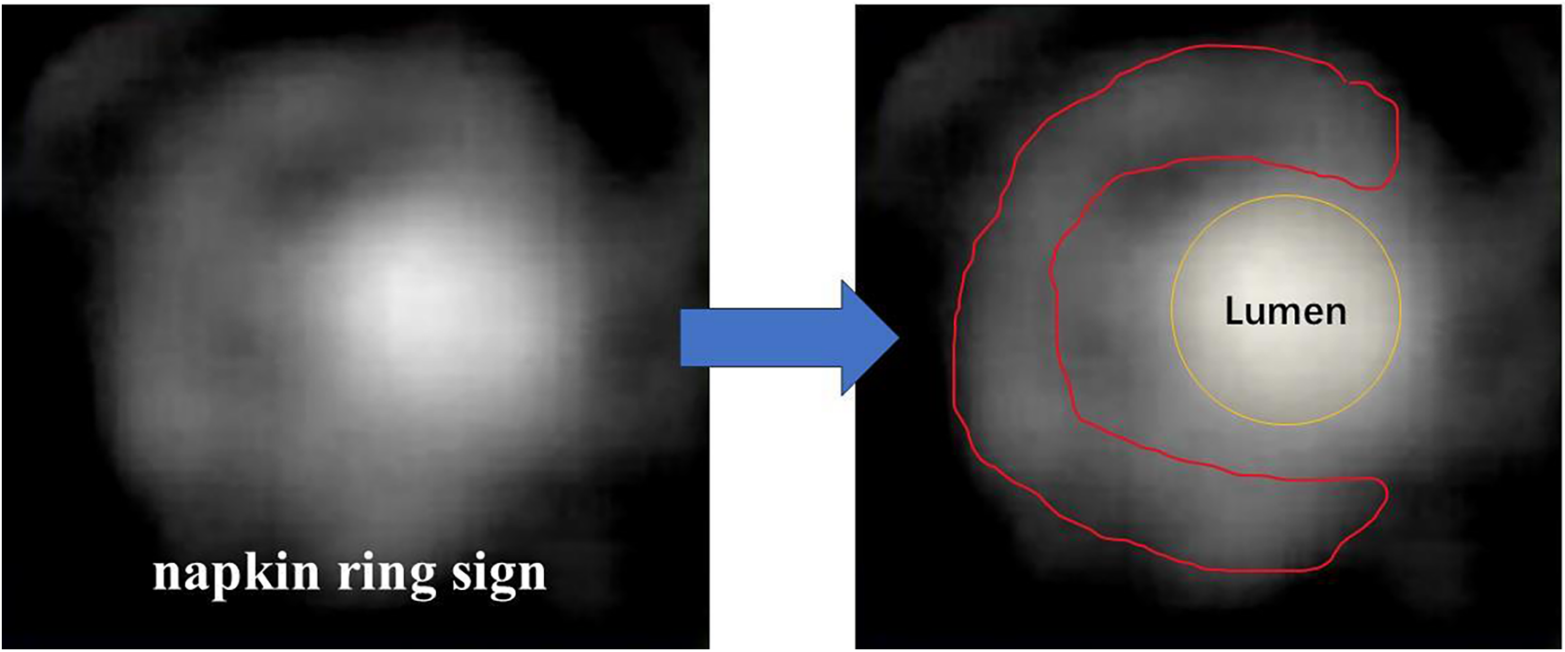
Typical images of napkin ring sign. Lumen contour (yellow line) and fibrous tissue contour (red line) are shown.

While non-contrast CT is limited to quantifying the burden of non-calcified and vulnerable plaques or stenosis, patients can have the intravenous administration of iodine contrast media to get more information (8). Different from conventional angiography, computed tomography angiography (CTA) enables better visualization in three dimensions by the reconstruction and identification of certain characteristics of vulnerable plaques, such as positive remodeling, low-attenuation plaque, napkin ring sign (9) (Figure 1) and spotty calcification (Table 1). Multi-directional imaging with a relatively high spatial resolution (e.g., up to 0.23 mm spatial resolution of last-generation CT scans) and fast scanning speed is suitable for the observation of early vascular lesions that are not easy to find. In the Scottish Computed Tomography of the HEART (SCOT-HEART) trial, compared with standard care, CTA reduced the clinical outcomes-related secondary endpoint of the rate of cardiovascular death or myocardial infarction at 20 months, and increased diagnostic certainty and the frequency of a diagnosis of coronary heart disease at 6 weeks (10). In the PROMISE trial, the investigators also reported that CTA was associated with a 34% relative reduction in all-cause death and myocardial infarction at 12 months (10).

**Table 1:** CT signs of high-risk plaques.

Signs	Characteristics
Positive remodeling	The width of the outer wall of the vessel is greater than 10% of the proximal and distal reference values of the plaque.
Low-attenuation plaque	The plaque CT value is less than 30 HU.
Napkin ring sign	The low-density component of the plaque is surrounded by a high-density ring of contrast medium, together with the contrast agent in the lumen of the blood vessels.
Spotty calcification	Calcifications are distributed along the long axis of coronary arteries and less than 3 mm in size.

Atherosclerotic plaques with 2 or more of the above signs at the same time are defined as high-risk plaques for CT.

Clinical evidence indicates that the phenotype of perivascular adipose tissue (PVAT) is closely related to inflammation and metabolism of the adjacent vasculature, regarded as one of the pathogenesis of atherosclerosis (11). CTA is currently the main imaging modality for evaluating inflammation of PVAT. Recently, a CTA-based biomarker, the peri coronary fat attenuation index, detecting active vascular microcalcification or inflammation by analyzing the three-dimensional changes of CT attenuation from the PVAT around diseased artery segments, is considered a novel metric of inflammation (12, 13). Sun JT, et al. (14) designed a prospective study extending the findings to suggest that the pericoronary fat attenuation index may be a reliable indicator associated with plaque vulnerability. As a result, the fat attenuation index can significantly improve cardiac risk identification and prognosis in both primary and secondary prevention. Similarly, epicardial adipose tissue (EAT) has unique properties, which produce cytokines that nourish the heart in healthy conditions. However, chronic inflammatory disorders can lead to deranged adipogenesis causing atrial and ventricular fibrosis. While high-risk plaque burden identified through assessment of low-attenuation non-calcified coronary plaque volume on CTA was found to concern prognosis, Yamaura H, et al. (15) also proposed that EAT volume is independently associated with increased LAP volume. The combination of CACS and EAT volume provides a non-invasive method of identifying lesions that may develop severe coronary events.

Among the non-invasive imaging in the cardiovascular field, CT is widely used in clinical practice, such as measuring CACS in asymptomatic population and having a CTA scan in patients with acute chest pain and low (about 0–2) Thrombolysis in Myocardial Infarction risk score (5). However, due to its limited sensitivity and specificity, CT is not perfect. Although the CT imaging data are now available at low x-ray exposure, the radiation exposure should be considered as well. Moreover, as for CTA, the contrast medium needed also puts patients at risk of allergic reactions, contrast-induced nephropathy, and so on. Due to an insufficient imaging resolution, CTA has limited accuracy in identifying an intraplaque hemorrhage and a large lipid core and is poor in predicting plaque erosion, which is the potential culprit of about one-third of acute events (2).

### 2.2 Magnetic resonance (MR)

As is known, MR imaging has the advantages of multi-sequence imaging, multidirectional reconstruction, and excellent soft tissue resolution. While several high-risk plaque features can be detected in CTA as mentioned, MR can provide more detailed and comprehensive imaging. For example, magnetic resonance angiography can accurately detect thin fibrous caps which is difficult to discriminate in CTA (16, 17). The multiple sequences of MR give it a great advantage in distinguishing plaque components. In non-contrast T1-weighted sequences, for instance, lipid-rich necrotic plaques, or intraplaque hemorrhage, displayed on T1-weighted sequences tend to have short T1 values, that is, high signal intensity. Therefore, high-intensity plaques are regarded as vulnerable lesions.

Currently, vessel wall high-resolution magnetic resonance imaging (VW-HRMRI) plays an important role in early diagnosis and clinical treatment. There is high consistency between VW-HRMRI and histopathology in detecting vulnerable plaques, such as qualitative analysis of the characteristics of intraplaque hemorrhage, intraplaque inflammation and neovascularization, plaque surface ulceration, positive vascular remodeling, lipid-rich necrotic cores and thin fibrous caps, and quantitative analysis of common parameters include plaque volume and thickness, the normalized wall index, the remodeling index and the vascular stenosis ratio, and so on (3). However, VW-HRMRI is limited to a single plaque or vascular segment, challenging the evaluation over larger sections of the arterial tree.

MR based on gadolinium, of which the accumulation in the arterial wall is correlated with endothelial permeability and inflammation, can provide better spatial resolution. Plasma concentrations of myeloperoxidase (MPO) can predict future adverse coronary events and are most likely associated with plaque instability (18). Gadolinium-enhanced MR imaging has been shown to non-invasively distinguish increased MPO activity in vulnerable plaques (19); thus it has the potential to identify high-risk plaques and evaluate therapies. However, the contrast agent based on gadolinium chelates is concerned with systemic toxicity and deposits. To address the concerns, Evans RJ, et al. (20) developed a non-toxic and high relaxivity probe based on superparamagnetic iron oxide nanoparticles, which accumulate in similar regions to an elastin-targeted agent, for visualizing plaque burden. Although the novel contrast agent is not specific for vulnerable plaques, it may contribute to improving specific contrast agents in the future. What's more, with the advancement of nanotechnology, more and more attention is paid to the development of targeted medical imaging and therapeutic strategies. Bagalkot V, et al. (21) reported lipid–latex hybrid nanoparticles selectively targeting inflammatory macrophages like plaque M1 macrophages and releasing a load of therapeutic materials like anti-inflammatory drugs. Though the nanoparticles cannot pass regulatory requirements for clinical translation, the principles of phagocytic targeting can be generalized to any other biocompatible and biodegradable nanoparticles. Foamy macrophages can secrete a large number of inflammatory cytokines to play a role in the development and rupture of atherosclerotic plaques which cause the vulnerability of plaques. Among inflammatory cytokines, IL−6 is thought to be possibly used as an independent marker for predicting future cardiovascular events (22). Mo H, et al. (23) prepared IL-6-targeted superparamagnetic iron oxide nanoparticles synthesized by a chemical condensation reaction and demonstrated its IL-6-targeted characteristic, as well as the usefulness for detection of vulnerable plaques with good sensitivity, specificity, and biocompatibility. Another example is that Dai Y, et al. (24) modified Gd-doped Prussian blue with polyethyleneimine, rhodamine, and dextran sulfate via electrostatic adsorption, which reduced inflammation, apoptosis, and the formation of foam cells.

Compared with CT, MR has certain advantages including better evaluation of soft tissue, lack of blooming artifacts, and no exposure to radiation, but its limitations, including a relatively long scan time for the assessment *in vivo* due to its high signal-to-noise ratio, and contraindications like patients with claustrophobia or metal devices, cannot be ignored either. Also, the low spatial resolution of MR (0.6 mm) makes it poor in clinical practice, for example, detection of coronary arteries is challenging for MR due to their small diameter, deep location, and influence by cardiac cycles and diaphragmatic movements. Without available data, the prediction of plaque erosion, just like CT, is worrying (2). Maybe the development of motion correction techniques and image reconstruction is expected to be better adapted to clinical applications in the future.

### 2.3 Non-invasive ultrasound

Ultrasound, as a widely used diagnostic tool in clinical practice, is portable, non-radiation, and relatively low-cost. Nowadays more novel ultrasound techniques have been developed, occupying an important position in the cardiovascular field. In addition to intravascular ultrasound imaging, non-invasive methods can also be used for the evaluation of vulnerable plaques. Table 2 summarizes the role of different non-invasive ultrasound in the detection of vulnerable plaques.

**Table 2:** An overview of the mechanism, advantages, and limitations of three common types of non-invasive ultrasound in the detection of vulnerable plaques.

Ultrasound method	Mechanism	Advantages	Limitations
B-Mode with/without Doppler	Display echo signals in grayscale, with or without the processing of blood flow signals according to the Doppler effect	• Contribute to cardiovascular risk assessment by measuring IMT • Provide information on flow velocity and stenosis severity with Doppler	• Low reproducibility • Limited exploration depth of vascular ultrasound • Trouble in detecting blood flow velocity less than 0.4 cm/s
Contrast-enhanced ultrasound (CEUS) and molecular imaging	Give Intravenous contrast to enhance the blood flow scatter signals or use microbubbles targeting the tissue or the organ	• A high pathological correlation • Detecting intraplaque neovascularization • Filling in the gaps such as improper insonation angle, low blood flow rate, deeper artery location • Microbubbles carrying molecular reagents to achieve the goal of theranostic applications in the molecular field	• Limited enhancement time • High technical requirements • No standardization
Elastography	Apply an internal or external dynamic/static/quasi-static excitation to analysis of the tissue in combination with digital signal processing or digital image processing techniques	• Semi-quantitative evaluation • Distinguishing lipid, fibrosis, calcification • Even higher specificity compared to CEUS	• Limited to linear behavior in an isotropic, semi-infinite medium • Affected by the pulsation of the vessel wall • Inter-observer and intra-observer variation

**Figure 2:**
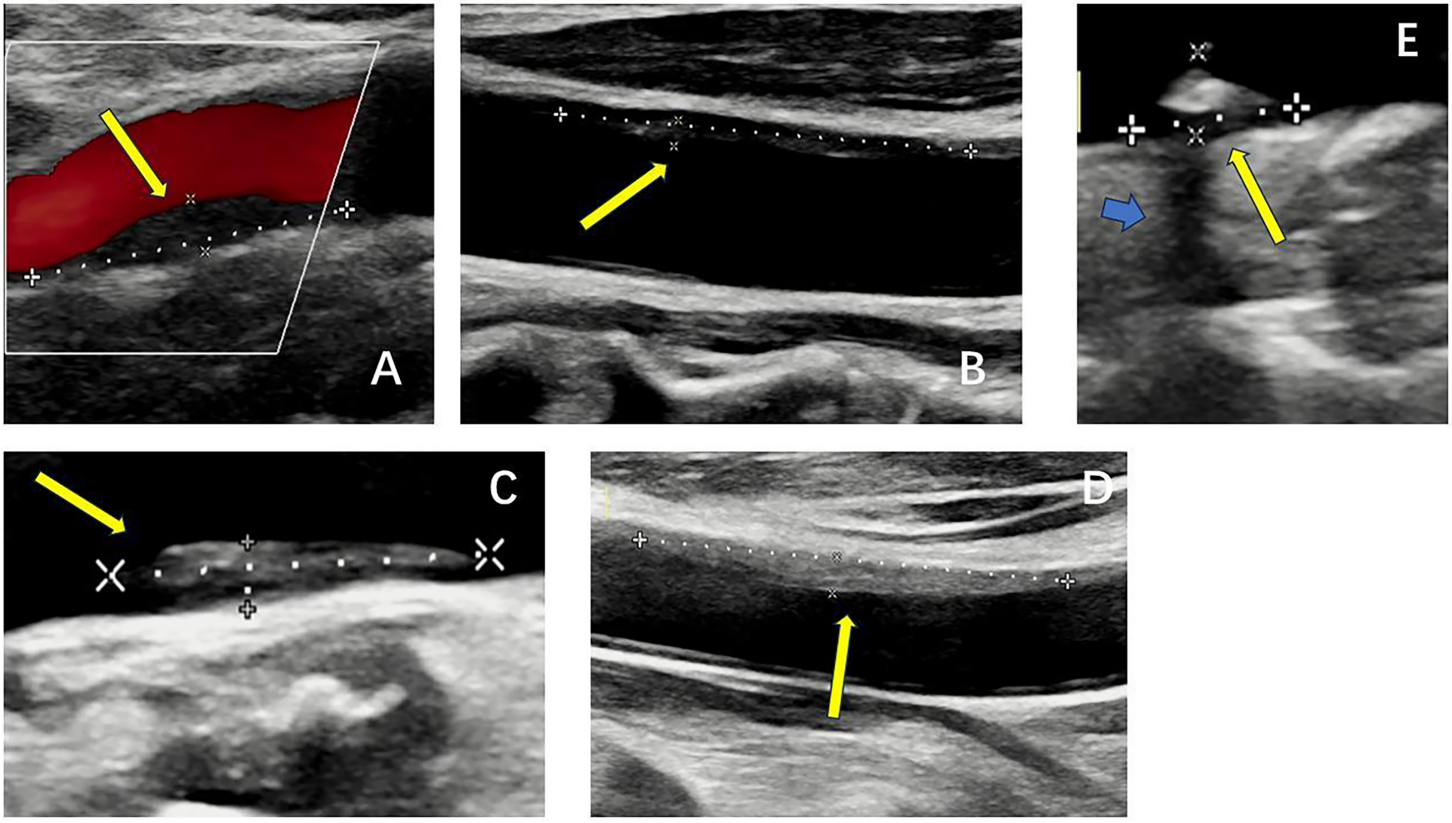
B-mode ultrasound images of five types of plaques distinguished by the gray-weale–nicolaides (GWN) classification including **(A)** uniformly echolucent plaque, **(B)** predominately echolucent plaque, **(C)** predominantly echogenic plaque, **(D)** uniformly echogenic plaque, and **(E)** heavy calcification. The yellow arrows indicate plaques. The blue arrow indicates the shadow.

**Figure 3:**
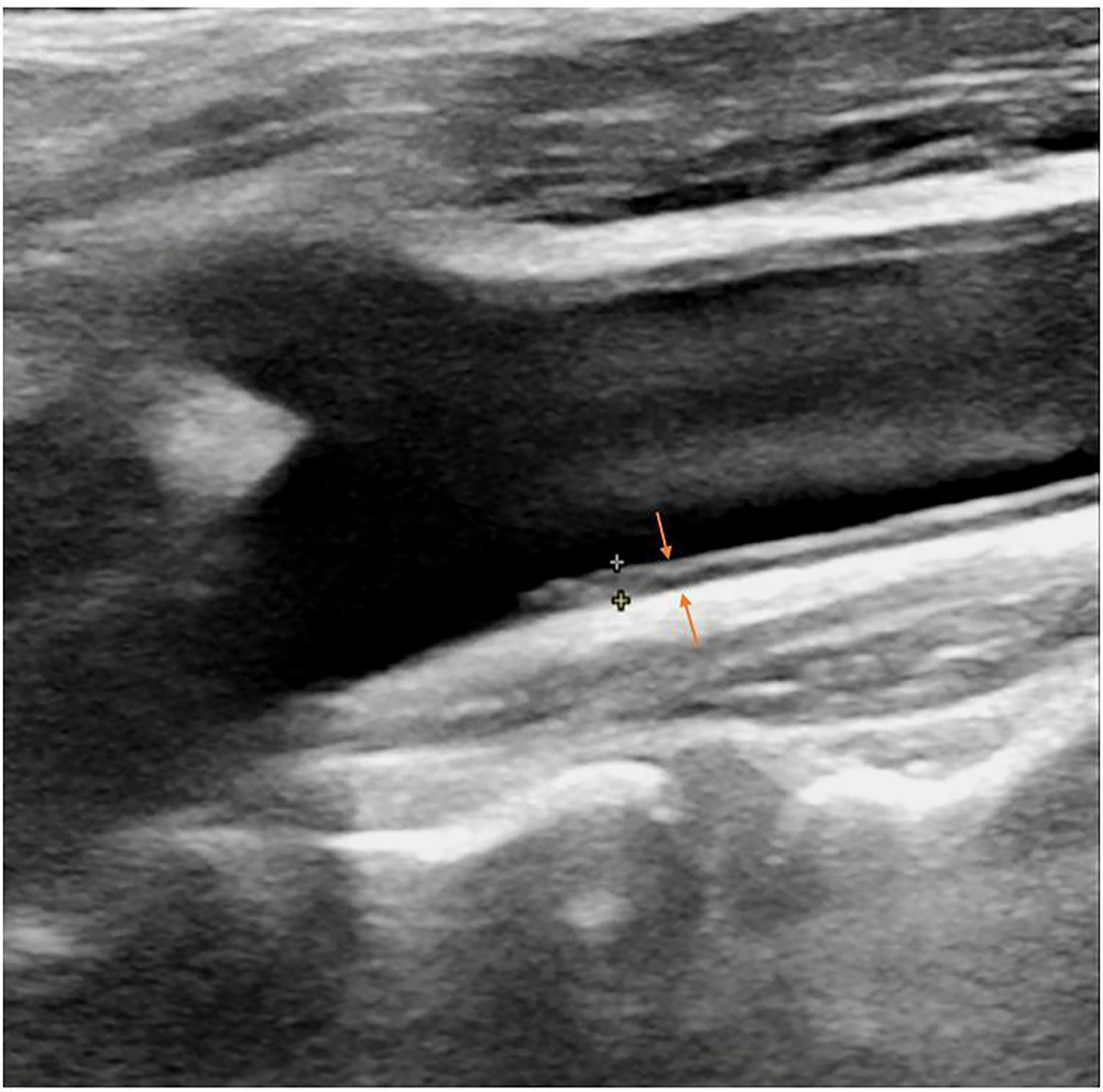
B-mode ultrasound image of carotid intima-media thickness (IMT) (arrows).

Real-time ultrasound (B-Mode, 2D Mode) is the primary imaging modality for initial assessment that can diagnose artery stenosis, as well as identify and locate plaque (25). With Doppler, color flow mode, it provides information on flow velocity, stenosis severity, and plaque surface and composition. There is an ultrasound-based classification, called Gray-Weale–Nicolaides (GWN) classification, distinguishing between five types (classes) of plaques, that is, uniformly echolucent plaque, predominately echolucent plaque, predominantly echogenic plaque, uniformly echogenic plaque, heavy calcification (26). (Figure 2) In addition, carotid intima-media thickness (IMT) is a quantitative parameter measured using high-resolution B-mode ultrasound, which is implicated in cardiovascular risk assessment. (Figure 3) IMT is more considered a surrogate marker of atherosclerosis, and yet its importance is rather debatable (27, 28). Also, considering the asynchronous movement of the plaque from B-mode ultrasound associated with a higher risk of stroke, Golemati S, et al. (29) statistically analyzed 135 plaques in 77 patients (59 men, 18 women) with carotid atherosclerosis and proved the ability to obtain tissue kinematic characteristics. It can be utilized to develop ultrasound-based risk stratification.

Due to the frequency of the linear-array probe (around 5–10 MHz), nevertheless, the exploration depth of vascular ultrasound is limited. To solve this problem, contrast agents (mostly microbubbles) can be used. At present, contrast-enhanced ultrasound (CEUS) is commonly used in clinical practice for defining endocardial border or for left ventricular opacification. When used to assess atherosclerotic plaques, CEUS compensates for defects such as improper insonation angle, low blood flow rate, deeper artery location, and difficulty in identifying intraplaque neovascularization with conventional B-mode ultrasound, and even allows for better measurement of IMT (30, 31). A study confirmed that CEUS has a high pathological correlation (32). The novel technology is also expected to achieve the goal of theranostic applications in the molecular field. VCAM-1 is a promising target for the detection of early and advanced atherosclerotic lesions as well. The use of microparticles for detecting VCAM-1 in molecular imaging methods has been described (31, 33). Since VCAM-1 is located on the endothelial surface, the blood contrast agent is easily accessible. However, to date, studies have not been tested outside of animal models, and the clinical applicability remains a challenge. Moreover, molecular expression varies at different stages of atherosclerosis development, making it impossible to obtain complete information from a single targeted imaging.

Besides, elastography can translate the obtained elastic information of biological materials into visible images so that we can distinguish the mechanical properties of tissue materials and then determine the possible pathological changes, position, shape, and size of corresponding tissues or organs based on their conditions. Using the principle of elastography, we can distinguish components of plaques like lipid (soft), fibrosis (moderately hard), calcification (hard), etc. Given that stable plaques are fibrocalcific, heterogeneous strain distribution suggests plaque vulnerability. In addition, neovascularization may improve the performance of ultrasound non-invasive vascular elastography, which possibly makes it a valuable alternative to MR for plaque assessment (30). Strain and shear-wave elastography allows for semi-quantitative evaluation of plaques and the characterization of plaque components. Shear-wave elastography, a common measure to quantify elastic modulus distribution in atherosclerotic plaque, seems more unequivocal than strain elastography (34). What's more, Di Leo N, et al.’s (35) research reflected high sensitivity (87.1%) of shear-wave elastography and even higher specificity compared to CEUS (66.7% vs.58.3%) in identifying vulnerable plaques. However, assumptions of linear behavior in an isotropic, semi-infinite medium and the pulsation of the vessel wall limit its application. More extensive and long-term controlled clinical studies are required to further validate the accuracy of elastography in detecting vulnerable plaques.

### 2.4 Positron emission tomography (PET)

Inflammation is a major factor in the development and progression of atherosclerosis. In recent years, the assessment of plaques has gradually shifted from a single morphological analysis to a comprehensive evaluation of morphological characteristics and inflammatory activity. With molecular imaging being developed, PET imaging, a non-invasive nuclear imaging technique, is at the forefront. Based on the employing intravenous administration of a selective radiotracer, PET imaging can target specific biochemical processes *in vivo*. Based on its characteristics, PET imaging has been widely used in oncology and neurology. It has recently been considered for clinical applications such as risk stratification and prediction in the cardiovascular field because of its highly sensitive assessment of disease activity. To achieve this goal, the most important thing is to develop the right radiotracer.

The use of glucose analogs like ^18^F-fluorodeoxyglucose (^18^F-FDG) is most common, which targets the inflammation that relies on the highly metabolic state of macrophages, and takes advantage of the characteristics of abnormal glucose metabolism in the lesion for localization, diagnosis, and evaluation. Galiuto L, et al. (36) demonstrated for the first time that there is a high correspondence between increased ^18^F-FDG uptake and the unstable morphology of coronary plaques on intravascular imaging. However, it should be noted that due to its non-specificity, it can also be taken up by adjacent tissues limiting imaging accuracy, especially in the myocardium, which has high glucose metabolism and high affinity for ^18^F-FDG (37). It is necessary to perform special patient preparation in advance to minimize myocardial glucose uptake (4, 38, 39).

In addition to inflammation, microcalcification, which creates local mechanical stress on the plaque surface leading to plaque rupture, is also associated with vulnerable plaques (40). PET imaging detects microcalcification more reliably than other non-invasive imaging modalities. Therefore, several recent studies are exploring the application of ^18^F-sodium fluoride (^18^F-NaF) in detecting vulnerable plaques. ^18^F-NaF is a positron bone imaging agent. It is concentrated by bone tissue through ion exchange, reflecting changes in bone metabolism. Interestingly, it tends to bind to newly formed hydroxyapatite more than the old crystals, which is more helpful in identifying actively inflamed calcifications (4, 41). What's more, ^18^F-NaF may detect areas of tissue necrosis at the site of myocardial infarction and even be related to PVAT, another marker of vascular inflammation mentioned above (37). A combination of features suggests that it is likely to be an excellent choice for the detection of vulnerable plaques.

Besides, there are various other targeted tracers for the detection of atherosclerosis. It is generally believed that nicotinic acetylcholine receptors (nAChRs), pentameric ligand-gated cation channels, are mainly found in central and peripheral nervous system diseases. Recently, however, it has been found that nAChRs, especially the α7nAChRs, can also be expressed in endothelial cells and may be an early target for vulnerable atherosclerotic plaques. Wang D, et al. (42) verified the reliability of an ^18^F-labeled PET molecular probe with high selectivity for α7nAChR in early diagnosis of atherosclerosis, which may play an early warning role in cardiovascular events. Further studies on the radiotracers for early plaque diagnosis are ongoing. Equally, ^68^Ga-pentixafor is thought that plaque vulnerability can be assessed through cell recruitment, the accumulation of oxidized LDL, and the formation of foam cells, which are related to Chemokine receptor-4, but fail to prove the exact cellular source (43, 44). Increased ^68^Ga-DOTATATE uptake with specificity for the G-coupled receptor somatostatin receptor subtype-2, whose expression is highly up-regulated in activated macrophages affected by an inflammatory stimulus, is associated with risk factors of cardiovascular disease (45, 46). More information on the different tracers is provided in Table 3.

**Table 3:** Examples of radiotracers in the identification of vulnerable plaques.

	Targets	Radiotracers	Characteristics	Limitations	References
Inflammation	Glucose metabolism	^18^F-FDG	Uptake in macrophage-rich areas of human plaques	Non-specificity	(47)
	18 kDa translocator protein (TSPO)	^11^C-PK11195	• In macrophage-rich regions • Lower background myocardial cell uptake • Distinguishing between recently symptomatic and asymptomatic plaques	Conflicting outcomes from previously reported TSPO PET clinical trials	(48)
	Microcalcification	^18^F-NaF	• Reliably detecting microcalcification and areas of tissue necrosis in the infarcted heart • Correlation with peri-coronary adipose tissue density	Inconsistency between accumulation of ^18^F-NaF and localization of arterial plaque	(41, 49)
	Chemokine receptor 4 (CXCR4)	^68^Ga-pentixafor	• High levels of ^68^Ga-pentixafor uptake correlated with cardiovascular risk factors • Insightful into cardiovascular risk and identifying early lesion development	Noisier images and worse spatial resolution	(43, 44)
	Somatostatin receptor subtype 2 (SSTR2)	^68^Ga-DOTATATE	• High specificity for the G-coupled receptor SSTR2 that is up-regulated in activated macrophages	Lack of further testing in a larger patient cohort	(45, 46)
	Folate receptor-β (FRβ)	^18^F-FOL	• Uptake correlated with macrophage density • No issue of high myocardial uptake	Lack of comparison between unstable and stable carotid plaques	(50)
Angiogenesis	αVβ3 integrin	^18^F-Galacto-RGD	Significantly higher radiotracer target-to-background ratios in stenotic compared with non-stenotic areas	Lack of larger prospective studies to evaluate the potential of molecular imaging of αvβ3 expression for assessment of plaque inflammation in patients	(51)
	α7 Nicotinic acetylcholine receptor (α7nAChR)	^18^F-ASEM ^11^C-NS14492 ^18^F-NS14490	α7nAChR plays an exact role in atherosclerosis including the formation of foam cells	No radiotracer assessed in atherosclerosis	(42, 52)
Intraplaque hemorrhage	glycoprotein (GP) IIb/IIIa complex	^18^F-GP1	• Identifying acute arterial thrombus • A favorable biodistribution/kinetic profile	No follow-up data to discriminate between true or false-positive uptake	(53)
Hypoxia	–	^18^F-FMISO	Uptake in symptomatic carotid plaques (due to hypoxia correlated with adverse features of plaque biology, including inflammation and intraplaque hemorrhage)	The limited sample size	(54)
Apoptosis	–	^18^F-ML-10	Successfully detecting apoptosis in a rabbit model with atherosclerotic plaques rich in apoptotic cells	Lack of research on the specificity of vulnerable atherosclerotic plaques	(37)

Though PET has advantages in its excellent sensitivity and quantitative efficiency, disadvantages such as the low spatial resolution (about 6 mm), the effects of heartbeats and respiration on signals, radiation exposure, and high cost cannot be ignored. Since PET signals cannot distinguish between plaques with rupture and plaques with erosion, it may not be suitable to provide adequate indications for individualized treatment. At the same time, due to the shortcomings of PET in anatomical structure, it also needs to be combined with CT or MR to obtain a more accurate diagnosis.

The non-invasive imaging modalities described above have their advantages and disadvantages and are generally less hurt. The comparison among them is summarized in Table 4.

**Table 4:** Comparison of different non-invasive imaging methods in the detection of vulnerable plaques.

Modalities	Plaque components/features	Advantages	Limitations	Recent progressions
CT (e.g., non-contrast CT and CTA)	• Calcification (e.g., CACS) • Positive remodeling • Low-attenuation plaque • Napkin ring sign • Spotty calcification	• Reconstruction • Relatively high spatial resolution • Fast scanning speed	• Limited sensitivity and specificity • Radiation exposure • Risk of allergic reaction, contrast-induced nephropathy, and so on (by CTA) • Limited accuracy in identifying an intraplaque hemorrhage and a large lipid core • Poor in predicting plaque erosion	• The main imaging modality for evaluating inflammation of PVAT (e.g., a CTA-based biomarker, FAI) and EAT • Functional assessment (e.g., myocardial CT perfusion or non-invasive CT-derived fractional flow reserve)
MR (e.g., MRA, HRMRI, non-contrast T1-weighted sequences, and contrast-enhanced MR)	• Thin fibrous caps • Lipid-rich necrotic plaques (high-intensity plaques displayed on T1-weighted sequences) • Intraplaque hemorrhage (high-intensity plaques displayed on T1-weighted sequences)	• Multi sequence • Multidirectional reconstruction • Excellent soft tissue resolution • Lack of blooming artifacts • No exposure to radiation	• Relatively long scan time due to a high signal-to-noise ratio • Contraindications (e.g., claustrophobia and metal devices) • Low spatial resolution • Concerned with systemic toxicity and deposits (by MR based on gadolinium) • Poor in predicting plaque erosion	• High consistency between VW-HRMRI and histopathology in detecting vulnerable plaques • Non-invasive imaging of plaque MPO activity • Hybrid nanoparticles and nanocomposites (21, 23, 24)
Non-invasive ultrasound (e.g., B-mode ultrasound with/without Doppler, CEUS, and elastography)	• IMT • Intraplaque neovascularization (by CEUS) • Flow velocity, stenosis severity, and plaque surface and composition • Distinguish components of plaques like lipid, fibrosis, calcification, etc. (by elastography)	• Portable • Relatively low-cost • No exposure to radiation	• B-mode ultrasound: the limited exploration depth and resolution • CEUS: limited enhancement time and high technical requirements • Elastography: still requiring more extensive and long-term controlled clinical studies	• CEUS and theranostic applications in the molecular field (e.g., a promising target, VCAM-1) • Strain and shear-wave elastography
PET	• Inflammation (e.g., microcalcification) • Intraplaque hemorrhage • Neovascularization	• Highly sensitive assessment of disease activity • Excellent quantitative efficiency	• Low spatial resolution • Affected by heartbeats and respiration • Radiation exposure • High cost • Poor in providing anatomical structure	Novel radiotracers in the identification of atherosclerosis (e.g., ^18^F-NaF)

## 3 Invasive imaging

Invasive imaging, such as optical coherence tomography (OCT), intravascular ultrasound imaging (IVUS), and near-infrared spectroscopy (NIRS), enables the accurate assessment of vulnerable plaques in high-risk patients.

### 3.1 Optical coherence tomography (OCT) and intravascular ultrasound imaging (IVUS)

Based on the principle of low coherence interference, OCT can reconstruct a two-dimensional or three-dimensional image of the internal structure through scanning. The signal contrast is derived from the spatial variation of the optical reflection/scattering characteristics. In terms of imaging, the fast data acquisition speed and lack of artifacts make it an excellent advantage as a potentially invasive tool for plaque detection. A comparative study has also confirmed the ability of OCT to assess vascular disease (55). Prati F, et al. (56) designed the first OCT study that directly found the morphology of coronary plaques obtained by OCT has the potential to be a powerful tool for predicting the risk of adverse cardiovascular events. The combination of multiple characteristics of OCT high-risk plaques is more useful in screening patients at higher risk of acute coronary events. Similar results were reflected in the Massachusetts General Hospital Optical Coherence Tomography Registry (57) which indicated lipid-rich plaques detected by OCT were at greater risk of non-culprit lesion-related major adverse cardiovascular events. At the same time, lesions of thin-cap fibroatheroma, a precursor lesion for plaque rupture, detected by OCT have also been shown to be associated with an increased risk of major adverse cardiovascular events during long-term follow-up (58). OCT is also the only imaging modality that accurately diagnoses plaque erosion *in vivo*. OCT-defined plaque erosion is divided into definite erosion and probable erosion (2, 59). The former has a thrombus covering the intact plaque, while the latter may be non-thrombotic but have an irregular luminal surface at the lesion, or the thrombus may attenuate the underlying plaque without superficial lipids or calcifications proximal or distal to the site. Furthermore, Huang M, et al. (60) compared the differences between the multilayer and single-layer 3D thin-layer models and found the single-layer model may overestimate the outer wall stress and underestimate the outer wall strain as well as the risk of plaque rupture. The current literature is affected by the lack of multilayer image segmentation technology and limited resolution of imaging modalities, therefore, the multilayer plaque model based on *in vivo* images has not been studied. However, the emergence of OCT has brought about an improvement in resolution, making it possible to distinguish the three-layer structure of blood vessels.

**Figure 4:**
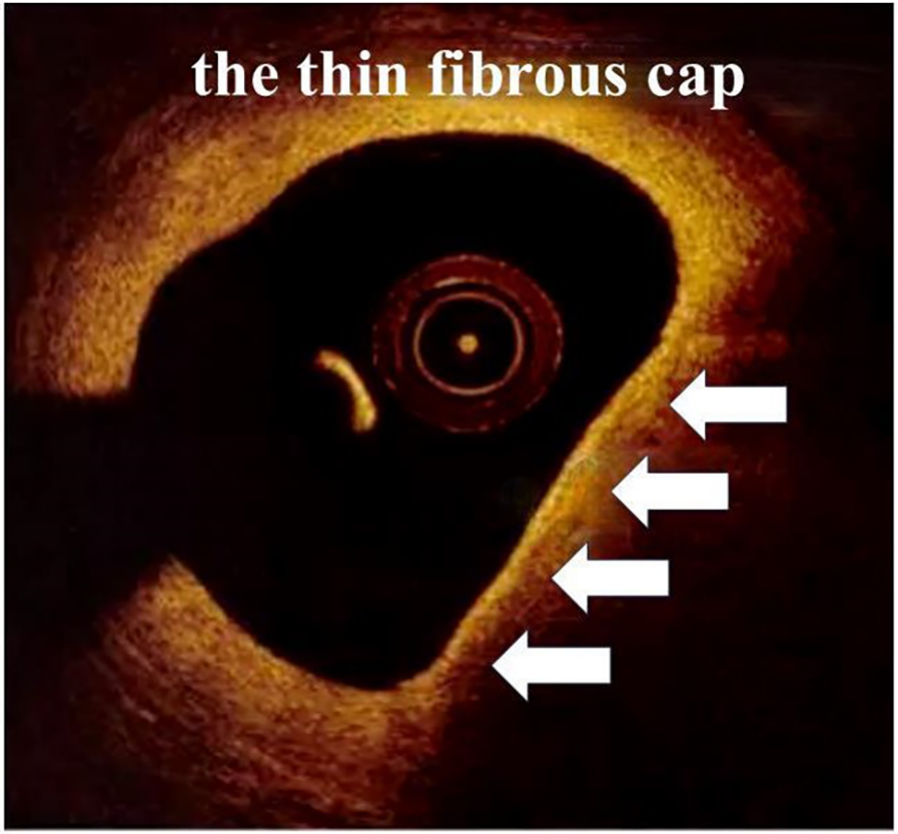
Typical OCT image of the thin fibrous cap (arrows).

When it comes to detecting atherosclerotic plaques, the high resolution (10–20 µm) of OCT allows it to discriminate superficial plaque components at a microscopic level, including quantifying the presence of macrophages and cholesterol crystal, discriminating lipid-rich plaques and visualizing fibrous cup thickness (Figure 4) and calcifications. However, it is limited by the poor penetration (1–2 mm) causing it not suitable for the measurement of plaque load. Unlike OCT, IVUS has greater penetration and can assess the entire arterial wall (38).

IVUS, utilizing a combination of non-invasive ultrasound technology and invasive catheter technology to image, is the first intravascular imaging modality to be applied in clinical practice. It visualizes the cardiovascular cross-sectional morphology and/or blood flow patterns, which can be used to examine the inner wall of the blood vessels. Grayscale IVUS can broadly divide plaques compared to surrounding adventitia echogenicity into four categories: soft plaques, fibrous plaques, calcified plaques, and mixed plaques (31). However, due to the distortion, grayscale IVUS cannot accurately discriminate plaque components. With the advancement of technology, intravascular ultrasound radiofrequency, i.e., virtual histology intravascular ultrasound (VH-IVUS), has emerged. VH-IVUS optimizes the differentiation of plaque components, thereby dividing plaques into fibrous tissue (dark green), fibrofatty tissue (light green), necrotic core (red), and dense calcium (white) (38). Additionally, high-definition IVUS facilitates the analysis of the luminal surface of blood vessels, which is expected to be used to detect plaque erosion (2). Generally, IVUS can provide qualitative assessments including identification of thin fibrous caps and analysis of plaque composition, as well as quantitative assessments such as plaque thickness, cross-sectional area, plaque burden, and remodeling index.

Whereas the insufficient spatial resolution (around 100–150 µm) of IVUS, the accuracy of the assessment of plaque composition is affected. Positive predictive values were found to be low at follow-up (61, 62). In a study of patients with ST-elevation myocardial infarction (61), VH-IVUS was found to be likely to overestimate the presence of thin-cap fibroatheroma probably because of its lack of the resolution to directly image fibrous cap thickness. The natural history Providing Regional Observations to Study Predictors of Events in the Coronary Tree (PROSPECT) study reported that IVUS was not able to visualize the entire coronary tree (63). Its resolution is proportional to the center frequency, but increasing the center frequency decreases the penetration depth, which can be improved if the probe can choose from a variety of frequency options depending on the region of interest (64). Due to the limitations of technology and process, nevertheless, the acoustic stack used for research is too large to perform intravascular application. Therefore, it still cannot be applied in clinical practice today.

The imaging signal of IVUS is susceptible to calcified areas, which makes it difficult to match OCT in terms of calcification. OCT can penetrate the calcified area of plaque, facilitate visualization of the adjacent tissue, and take a detailed measurement, dividing plaque calcifications into macrocalcifications, spotty calcifications, and microcalcifications (4). Unfortunately, OCT is inferior in detecting positive remodeling or plaque burden (61). In addition to the lack of clinical studies to establish a reliable clinical utility for predicting risk stratification, attenuation of light by blood also significantly impacts imaging and assessment. To overcome blood interference, methods such as saline flushes and balloon occlusion can be employed. Optical frequency domain imaging (OFDI) is a next-generation OCT technology that eliminates the need to block blood flow and provides fast imaging. Polarimetric measurements simultaneously with conventional OFDI cross-sectional imaging can obtain birefringence and depolarization of tissue (65). The high birefringence of the tunica media, as well as the significant depolarization of the lipid and necrotic core materials, make it not only provide a reliable assessment of the external elastic membrane but also determine fibrous cap thickness quantitatively and automatically. The first-in-human pilot study of intravascular polarimetry has also shown that caps of vulnerable plaques including ruptured caps exhibited lower birefringence than caps of stable lesions (66).

To sum up, OCT does better in imaging luminal plaque, calcium, and foreign body structures, such as stent struts in summary, while IVUS has a stronger penetrating ability and does not have the problem of being masked by blood flow, which has a greater advantage in vessels with larger luminal diameters.

### 3.2 Near-infrared spectroscopy (NIRS)

Because different tissues *in vivo* have different absorption and scattering characteristics of near-infrared light, the light has a strong ability to distinguish them. According to this characteristic, NIRS can be used to measure the optical parameters to obtain the physiological parameters of tissues, establish the relationship between physiological parameters and spectral data, generate two-dimensional images, or conduct clinical analysis. Recently, NIRS has been found to have the ability to detect high-risk plaques (67, 68). Plaques with a large lipid core tend to have a greater plaque burden, which is one of the risk factors for adverse cardiovascular events (69, 70). NIRS can accurately and quantitatively characterize the lipid content of a plaque based on the absorption pattern by cholesterol molecules, which is challenging in IVUS and OCT. Nonetheless, NIRS provides minimal anatomic visualization, that is, it is necessary to combine other modalities like IVUS to get more detailed and complete information on the lumen or the plaque (71). Lipid core burden assessment can be used as a supplement to plaque structure information for future use to refine risk stratification and treatment. On the clinical application of NIRS, there is relatively little clinical evidence, and more data are needed to support it.

### 3.3 Others

In addition to the invasive imaging modalities mentioned above, there are many other emerging techniques for detecting plaque features. Near-infrared fluorescence (NIRF) is a molecular intravascular imaging modality detecting inflammation, oxidative stress, and abnormal endothelial permeability with the help of fluorescent probes at a cellular and molecular level (72, 73), which may play a role in identifying plaques that are susceptible to erosion (2). Near-infrared autofluorescence can detect intraplaque hemorrhage by autofluorescence within the plaque (74). However, a clear association between NIRAF and cardiovascular disease is still needed through natural history studies with clinical follow-up. Besides, angioscopy visualizes the surface appearance of plaques like substantial information of atherosclerosis as yellow plaques (67), but has not demonstrated the clinical predictive value (74). A study performed survival intravascular photoacoustic imaging (IVPA) for quantitative assessment of lipid-rich atherosclerotic plaques in rabbits and demonstrated the potential of the clinical use of IVPA catheters for the detection of lipid-rich, high-risk plaques (75). Fluorescence lifetime imaging microscopy (FLIM) of plaques can measure the biochemical content near the luminal surface and evaluate the relative concentration of lipids and collagen.

Although the above imaging methods are invasive, the information obtained will be more accurate and intuitive. Table 5 compares the three main invasive imaging modalities (e.g., OCT, IVUS, and NIRS), and the advantages and disadvantages of the eight non-invasive and invasive imaging modalities mentioned in this paper in the visualization of vulnerable plaques are summarized (Table 6).

**Table 5:** Comparison of the three main invasive imaging methods in the detection of vulnerable plaques.

	OCT	IVUS	NIRS
Principle	Low coherence interference	Ultrasound and catheter technology	Near-infrared absorption wavelength and intensity
Penetration	Poor (1–2 mm)	Better (around 7 mm)	-
Resolution	High (10–20 µm)	Insufficient (around 100–150 µm)	-
Plaque Features	• Macrophages and cholesterol crystal • Lipid-rich plaques • Fibrous cap thickness • Calcifications • Plaque erosion	• Plaque thickness • Cross-sectional area • Plaque burden • Remodeling index • Thin fibrous caps • Entire arterial wall	Lipid content
Limitations	• Inferior in detecting positive remodeling or plaque burden • Blood interference • Lack of an appropriate risk assessment standard	• Low positive predictive values • Susceptible to calcified areas • Poor in visualizing the entire coronary tree	• Minimal anatomic visualization • Unable to be applied clinically alone

**Table 6:** Comparison of the eight non-invasive and invasive imaging modalities for visualization of vulnerable plaque. Among the modalities, NIRS needs to combine other modalities clinically, so the hybrid imaging mode of NIRS-IVUS is used here to add to the comparison.

** **	CT	MR	B-mode ultrasound	CEUS	PET	OCT	IVUS	NIRS-IVUS
Thin fibrous caps	−	+	−	−	−	++	+	+
Calcification	++	−	+	+	+	++	+	+
Neovascularization	−	−	−	++	+	−	−	−
Large lipid cores	−	+	−	−	−	++	−	++
Intraplaque hemorrhage	−	+	−	+	+	+	−	+
Inflammation	+	+	−	+	++	−	−	−
Positive remodeling	+	+	−	+	−	−	++	++

(−) modality is invisible or poorly detected; (+) modality is visible; (++) modality means suitable for detection.

## 4 Prospect

As the understanding of plaques has deepened, more and more attention has been paid to combining imaging modalities with different strengths to detect multiple features for a more accurate assessment of plaques, known as multimodality imaging. For instance, OCT with high resolution of thin fibrous caps can be fused with IVUS to accurately predict plaque cap thickness and obtain relevant mechanical information (76, 77). Nowadays co-registration of IVUS and OCT slices is performed using fiduciary points such as side branches, bifurcations, and calcifications. However, it is technically difficult to acquire both IVUS and OCT data at the same time. Data acquisition requires two separate catheterizations, both of which are invasive and expensive. The harm to the patient is undoubtedly greater (77). Devices that can perform both IVUS and OCT are under development and may be available shortly. Similarly, an emerging hybrid imaging modality combining IVUS with IVPA provides specific lipid detection and localization, and the dual-frequency IVPA/US catheter has a diameter of 1 mm, making it easy to accommodate current catheterization (78), while the OCT-NIRS catheter with automated co-registration of data contributes to improving stenting, as well as detect and treat vulnerable plaques (79). For the first time, Leng J, et al. (80) have developed a new multispectral intravascular three-modality imaging system integrating OCT, IVUS, and IVPA that can provide macro- and microstructural information of the vessel wall, as well as identify and quantify lipids. A single imaging method cannot provide comprehensive information about plaques, so multimodality imaging will be a promising future development direction, and clinical trials and technological breakthroughs with large samples are still needed.

The main focus of the discussion above is basically around the morphological imaging of vulnerable plaques. Recently, hemodynamic-associated biomechanical forces have also been taken into consideration. More than one study supports the close correlation between the formation and progression of plaques and hemodynamic characteristics, that is, the local hemodynamic parameters are likely to be regarded as prognostic markers for the assessment of plaque vulnerability (81–85). For example, the formation of plaque usually occurs in the low wall shear stress (WSS) area, while plaque instability is more related to high wall shear stress (81). The same results are also reflected in the research by Moerman AM, et al. (82) in which high time-averaged WSS is related to larger macrophage areas and necrotic core sizes. Their study is the first to show relations between the oscillatory shear index (OSI) and characteristics of plaque vulnerability, which supported low OSI is linked to larger macrophage areas as well, and a combination of low time-averaged WSS and low OSI is associated with larger cap thickness. In short, it contributes to the prognostic value of the prediction of plaques at different stages. WSS is currently measured invasively causing trauma to patients. Therefore, future development of similar algorithms in non-invasive imaging is required, like the CT-based measurement of fraction flow reserve. Computational fluid dynamics (CFD) is the most powerful research tool to assess patterns of WSS at present but needs to address many assumptions and procedures with strict requirements, such as three-dimensional reconstruction of the vessel, meshing, and solving the Navier–Stokes equation. Regarding the issue of three-dimensional reconstruction, coupled with a preference for non-invasive imaging, the trend is to combine the imaging system with CFD in the future (81, 83). Combining morphological and biomechanical features to determine plaque instability can improve diagnostic accuracy (86). In conclusion, morphological imaging with biomechanical measurements can better screen patients but still requires more clinical data support, and further improves algorithms as well as provides a set of standards.

## 5 Conclusion

In non-invasive imaging, CT, a high spatial resolution imaging method, has irreplaceable advantages in the evaluation of calcification and perivascular adipose tissue, but its radiation exposure and poor ability to distinguish between lipid and fiber components need to be paid attention to. In contrast, MR has no radiation and has a strong ability to identify soft tissues with multi-sequence and multi-directional reconstruction, so the plaque morphological information obtained is more comprehensive. However, its scanning time is too long, which affects the clinical application. The development of novel ultrasound techniques makes the assessment of plaque no longer limited to stenosis. Therefore, we can obtain more detailed information from the mechanical and even molecular aspects. PET, as a cutting-edge diagnostic tool for molecular application, can be used in the diagnosis of high-risk plaques as well.

In invasive imaging, the high resolution of OCT makes it accurate for the diagnosis of calcifications and thin fibrous caps and is the only imaging method for accurate diagnosis of plaque erosion *in vivo*. However, its penetration is not good and the signals are deeply affected by blood. IVUS, a commonly used intravascular imaging tool in clinical practice, can evaluate the lumen-related mechanical information and plaque burden. Different from OCT, its penetration of calcium is not high, and the resolution is still poor. NIRS has an excellent ability to detect lipids, but due to the limited anatomical structure information it provides, it still needs to be combined with other methods like IVUS in clinical use. In addition, there are many other emerging invasive imaging modalities such as NIRF, angioscopy, IVPA, and FLIM.

Despite the variety of imaging modalities available, none of them can be used alone to obtain comprehensive information to determine the vulnerability of plaques. Combining multiple imaging modalities to give full play to their respective advantages will be a significant trend in the future. At the same time, the combination of morphological and biomechanical characteristics of vulnerable plaques is also an important study. It is necessary to provide an evaluation standard for the combination of the two in the future, to make a more accurate early diagnosis of atherosclerotic plaques.
